# Preferential Paths of Air-water Two-phase Flow in Porous Structures with Special Consideration of Channel Thickness Effects

**DOI:** 10.1038/s41598-019-52569-9

**Published:** 2019-11-07

**Authors:** Jinhui Liu, Yang Ju, Yingqi Zhang, Wenbo Gong

**Affiliations:** 1 School of Mechanics and Civil Engineering, China University of Mining and Technology, Beijing, 100083 China; 2 State Key Laboratory of Coal Resources and Safe Mining, China University of Mining and Technology, Beijing, 100083 China; 3 Frontier Scientific Research Centre for Fluidized Mining of Deep Underground Resources, China University of Mining & Technology, 1 University Ave, Xuzhou, 221006 China; 4 Energy Geosciences Division, Lawrence Berkeley National Laboratory, Berkeley, California, CA94720 USA

**Keywords:** Hydrology, Geophysics

## Abstract

Accurate understanding and predicting the flow paths of immiscible two-phase flow in rocky porous structures are of critical importance for the evaluation of oil or gas recovery and prediction of rock slides caused by gas-liquid flow. A 2D phase field model was established for compressible air-water two-phase flow in heterogenous porous structures. The dynamic characteristics of air-water two-phase interface and preferential paths in porous structures were simulated. The factors affecting the path selection of two-phase flow in porous structures were analyzed. Transparent physical models of complex porous structures were prepared using 3D printing technology. Tracer dye was used to visually observe the flow characteristics and path selection in air-water two-phase displacement experiments. The experimental observations agree with the numerical results used to validate the accuracy of phase field model. The effects of channel thickness on the air-water two-phase flow behavior and paths in porous structures were also analyzed. The results indicate that thick channels can induce secondary air flow paths due to the increase in flow resistance; consequently, the flow distribution is different from that in narrow channels. This study provides a new reference for quantitatively analyzing multi-phase flow and predicting the preferential paths of immiscible fluids in porous structures.

## Introduction

Since the effect of preferential flow will be remarkable in leading the non-equilibrium fluid flow subsurface, accurate knowledge and description the preferential paths and interfacial dynamic of immiscible two-phase flow in rock mass and its influence factors are of great significance for the evaluation of oil or gas recovery^1,2^ and predication of roadway rock disasters^3^ due to the gas-liquid flow. Many studies have been conducted to analyze preferential flow in order to explore the influence factors including wettability^4–6^, capillary pressure^7,8^, injection rate^9^, viscosity ratio^6^ and capillary number^10^. However, few experimental and numerical studies are available for quantitatively and visually elaborating the fundamental processes of preferential flow in real rock structures through the effect of pore morphology and channel size, due to the complexity and heterogeneity of porous structures of natural rocks. Therefore, understanding and predicating the occurrence of preferential flow in porous structures, and its dependence on the pore morphology, channel width and thickness, is especially important, but fundamental knowledge regarding this topic is lacking. Most previous studies therefore relied on simplified models that neglected many of the important parameters of porous structures.

The spatially varying multiscale heterogeneity of rock mass makes two-phase flow modelling and path predication challenging inside reservoir rocks. The classical model considering a pore structure represented by a collection of random, evenly distributed spheres is no longer valid for studies on the fundamentals of flow behavior in real porous structures and must be replaced by other models. Lately, advanced imaging technologies have been used in laboratory to characterize the porous structures of rock mass, e.g. microtomography technology (MT) and X-ray CT scanning^11–14^. These porous structures are then etched into a transparent material, and tracer dyes and image analysis methods are used to visualize the flow pattern and track paths of fluid transport^15–21^. For instance, as early as in 1997, Keller *et al*.^18^ observed the flow of a non-aqueous phase liquid (NAPL, or oil), water, and air at the pore scale using a micromodel, providing direct observation of preferential path phenomenon for fluids traveling through porous structures. Chuang *et al*.^22^ characterize the preferential flow paths between boreholes in fractured rock using a nanoscale zero-valent iron tracer test, concluding that further detailed investigations on the preferential paths in real porous structures are required. Liu *et al*.^23^ used the X-ray microcomputed tomography to visualize the multiphase glow during core flooding experiments, and investigated the influence of phase morphology on relative permeability. Such laboratory methods are widely used in the simulation of two-phase flow in porous structures.

Two-phase fluid flow and flow paths in porous structures have also been extensively investigated numerically^24–31^ and attributed to explore the influence of a given factor on two-phase flow behavior. Zhang^7^ and Chang *et al*.^32^ showed that displacement processes with low capillary number and viscosity ratio are dominated by capillary pressure in numerical simulations. Zhang *et al*.^33^ characterized the preferential flow in cracked paddy soils using computed tomography. Al-Gharbi^34^ developed a dynamic pore-scale model for drainage displacement and imbibition processes observed in micromodel experiments. Compared to experimental studies, numerical simulation can more easily obtain the fluid flow pattern, capillary force and relative permeability in the real rock structures. In recent years, a new method called the Lattice Boltzmann Method (LBM) was widely used in studying multiphase flow, because it can implement boundary conditions to simulate the interface kinetics of fluids more effectively in complex porous structures^35–37^ without the requirements of the interface tracking method. Many LBM models have also been proposed in multi-phase flow in porous structures. Chen *et al*.^38^ used the color-fluid LBM model to simulate the process of liquid CO_2_ displacement in a two-dimensional heterogeneous micromodel at reservoir pressure conditions, and presented numerical simulation results of various capillary numbers and Reynolds numbers. This study visually and quantitatively compared the two-phase fluid flow patterns in a 2D heterogeneous pore model, and discussed the effects of fluid viscosity on the flow regime and flow path. Although the study by Chen *et al*.^38^ presented interesting results, the numerical simulations reported did not accurately reflect the experimental observations due to the limitations of numerical stability or the initial conditions. The fluid properties in most LBM models are severely limited, such that important variables such as two-phase fluid density, viscosity ratio constant, surface tension, fluid viscosity and lattice precision can only have a limited range of values. In the work of Chen *et al*.^38^, surface tension and fluid viscosity had values in a limited range, which may be one of reasons that the numerical simulation and experimental observations do not match. In addition, LBM models are also very expensive and it is necessary to perform long-term parallel calculations to simulate multiphase flow in porous media and obtain accurate results^39,40^.

Compared with LBM, the phase field method is directly based on the discrete partial differential equation of motion on the Euler grids and is preferable in multiphase flow because of its high numerical efficiency and ability to simulate multiphase fluid flow with larger density and viscosity ratio^41^. Phase field methods can be directly extended to pore-scale modeling, although the Navier-Stokes solver must work together with the phase interface method to track the two-phase interface^42^. For example, Basirat *et al*.^6^, used the phase field method to numerically investigate the two-phase flow of CO_2_ and brine at the pore scale, visually demonstrating that the wetting condition and viscosity ratio have obvious effect on the flow pattern and flow paths.

Minh *et al*.^43^ employed the phase field method to investigate the stability of a cracked rectangular plate with variable thickness. Schiedung *et al*.^44^ proposed a combined computational approach based on the multiphase field and the LBM for the simulation of capillary-driven kinetics. Although these last numerical studies investigated two-phase flow in different perspectives, these models assumed that perfect channel thickness is much smaller than the channel width without considering channel size effects. Little effort has been devoted to quantifying the influence of channel thickness on the occurrence of preferential flow. Furthermore, a direct comparison between simulated and experimental immiscible two-phase flow dynamic behavior in porous structures with various channel thickness has not been conducted, especially for the air-water flow with a larger density ratio (approximately 1000) and a high viscosity ratio (approximately 50) in complex heterogenous structures. In this work, a 2D phase field model capable of handling two-phase fluid interface kinetics of compressive fluids in heterogenous porous structures was built, and numerical simulations of air-water two-phase flow process in complex porous structures were conducted. This study attempted to explore explicit numerical analysis and experimental observations for understanding the fundamental processes of preferential flow in real porous structures. The effect of channel thickness on the interface dynamics of air-water flow and preferential paths in heterogeneous porous structures were examined. Simulations of flow paths yield results in line with experimental observations. Our analysis of experimental observations and numerical visualization of preferential paths reveals the main controlling factors on the preferential path of air-water two-phase flow. The influence of channel thickness on the air-water flow characteristics and flow path was revealed. This study presents an efficient numerical method for predicting the preferential paths in porous media and provides a new research reference for studying multiphase flow in rock structures.

## Results

In this section, we present the key results from the numerical simulations and experimental observations, focusing on displacement patterns, saturation, and flow paths. Results from both the numerical simulations and drainage experiments are shown, with both quantitative and qualitative comparisons made between them. Two different flow rates, namely, 3 ml/min and 10 ml/min corresponding to capillary numbers log Ca = −4.89 and log Ca = −4.38, respectively, were studied at ambient pressure and room temperature. These two cases are expected to fall within the crossover from capillary to viscous fingering and the viscous fingering regimes, respectively^1,7^.

### Flow regime and interface motion

**Figure 1:**
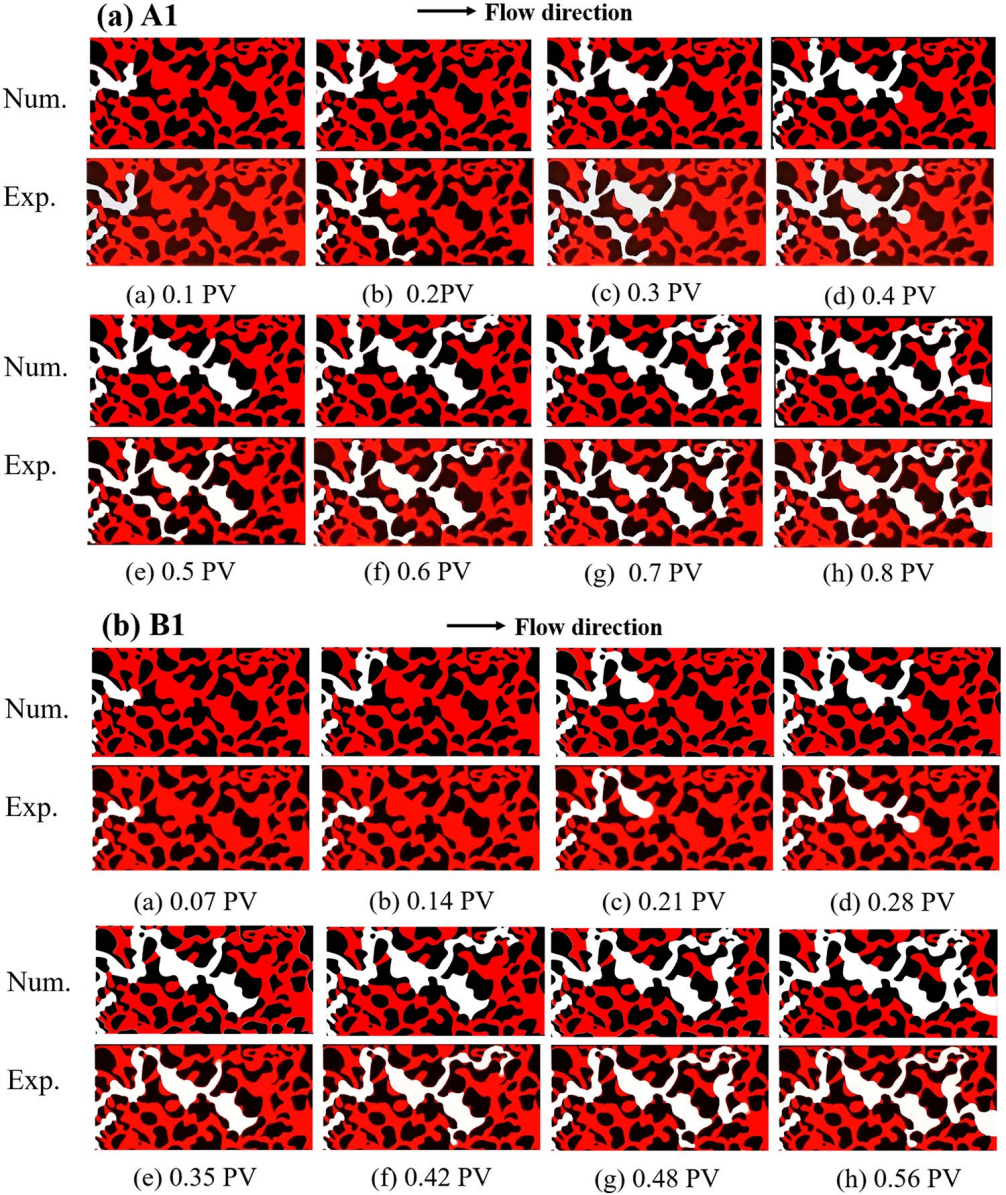
Snapshots of the fluid interface and flow paths for model A1 and B1. The top row shows the numerical simulations and the bottom row shows the experimental observations for different periods. The white areas represent the invading phase (air) and the red areas represent the displaced phase (water). The solid phase is shown in black.

The drainage displacement in porous media is the consequence of different paths and velocities experienced by water and air, involving the migration of the air front, and the evolution of individual menisci. Figure 1 plots the sketch of interface motions in the porous model with injected air pore volumes for two flow rates, from the numerical simulations and experimental observations. The results clearly show the water-air two-phase interfaces; red represents the resident water and white represents the air.

**Figure 2:**
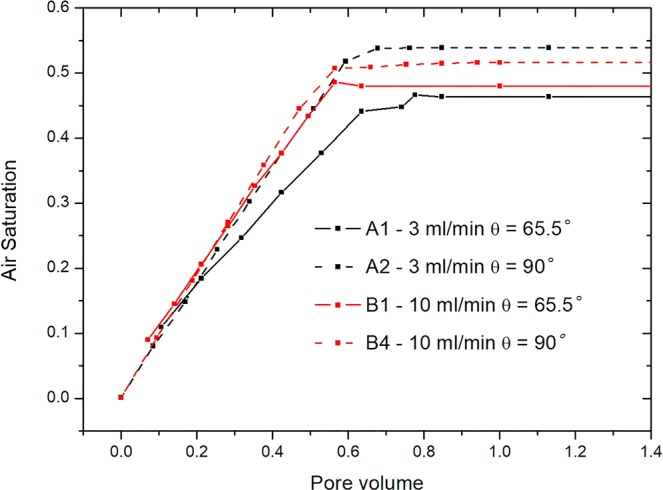
Air saturation variation with pore volume from the numerical simulations with various wetting conditions.

These snapshots of fluid interface from COMSOL accurately reflect the drainage displacement process. The entire drainage displacement process is primarily visible as the motion of air fronts until a breakthrough. Here, PV (Pore Volume) = 0 is chosen to correspond to the instant when the air front first reaches the porous section (i.e. when air saturation within the porous section first becomes non-zero). Figure 2 gives the variation of air saturation with the injected air pore volumes for various cases. These curves could plainly show the breakthrough time in each case. The rising portion of the air saturation curves represents the prime drainage period when the invading air phase principally enters the porous model.

### Flow velocity and flow paths

**Figure 3:**
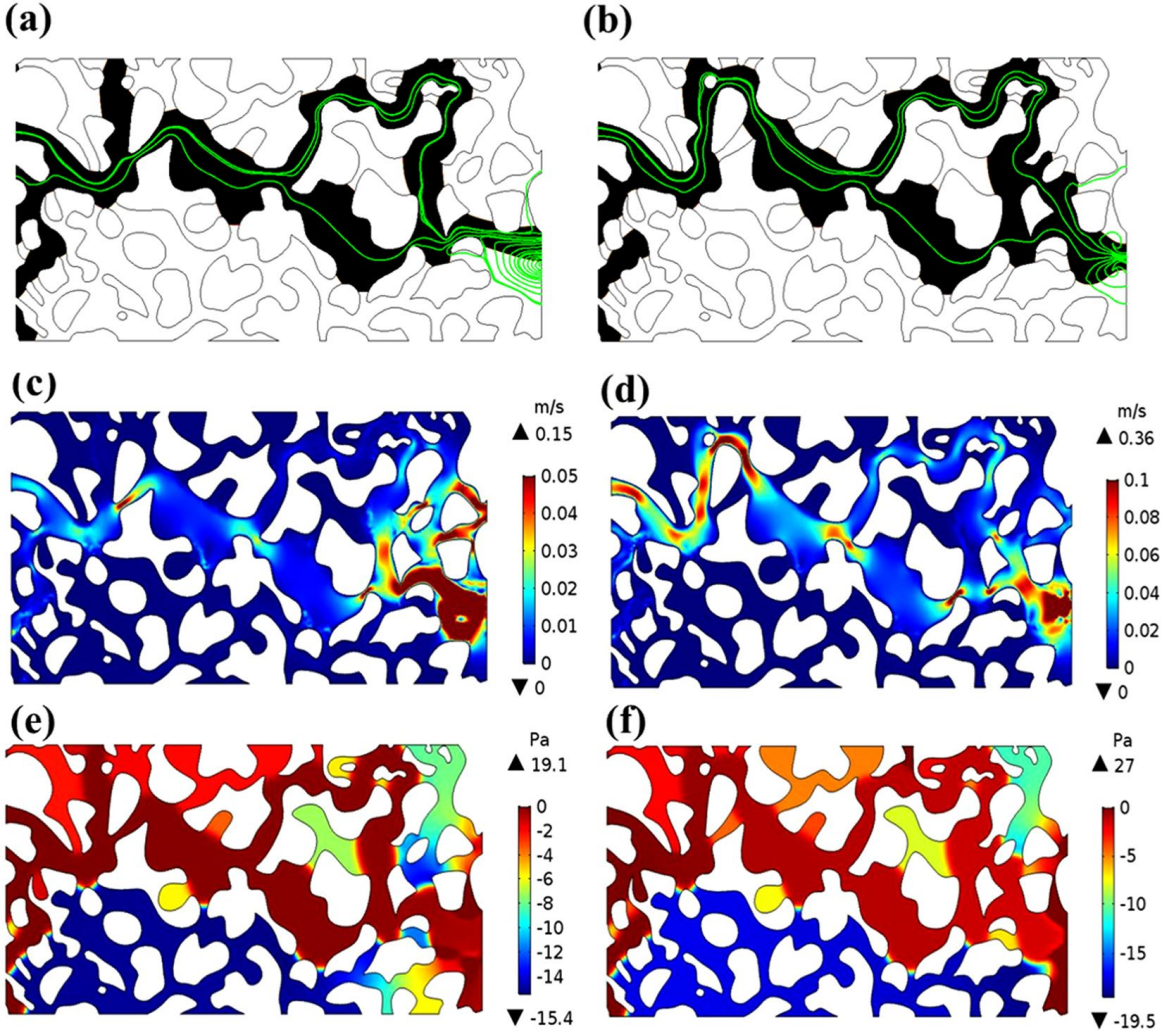
Flow paths, flow velocity distribution, and pressure profiles with pore volume from the numerical simulations with various flow rates. (**a**) Flow paths for A1; (**b**) flow paths for B1; (**c**) velocity distribution for A1; (**d**) velocity distribution for B1; (**e**) pressure profiles for A1; and (**f**) pressure profiles for B1.

Streamlines were used to track the preferential paths in the simulations. Figure 3 shows the flow paths represented by streamlines, as well as the velocity distribution and pressure profiles at the breakthrough time for two cases with different flow rates. It is shown that the main flow paths are followed by the fluids with the high flow velocity and high pressure drops, therefore, these two index distributions could well demonstrate the preferential paths in the porous model. By comparing Figs 1 and 3 we found that the velocity flow branches from the numerical simulation significantly overlap with the flow peak paths from the experiments.

Figure 3 shows that air first infiltrates into the porous section and it forms preferential paths. Air infiltrations occur simultaneously in multiple branches, and under a given injection velocity the preferential paths depend on the pore morphology of each branch channel. When the air front arrives at the pore throats, the wide channels are the main flow paths in a certain period despite of the existence of large pores behind tight channels, which indicates that the channel size is crucial to flow path selection. As the flow front reaches some tight throats, whether fluid can pass through can depend on the capillary pressure. The relationships between capillary pressure and saturation were widely used to describe the two-phase flow. This study defines the macro-scale capillary pressure, $\Delta {{\boldsymbol{P}}}_{{\boldsymbol{avg}}}$, as the difference between the intrinsic phase average of the non- wetting and wetting pressures^6,45,46^, formulated as follows:

1 $$\Delta {{\boldsymbol{P}}}_{{\boldsymbol{avg}}}={{\boldsymbol{P}}}_{{\boldsymbol{nw}}}-{{\boldsymbol{P}}}_{{\boldsymbol{w}}},$$

2 $${{\boldsymbol{P}}}_{{\boldsymbol{nw}}}=\frac{{\int }_{V}{\boldsymbol{p}}\,{V}_{f2}dV}{{\int }_{V}{V}_{f2}dV},\,\,{{\boldsymbol{P}}}_{{\boldsymbol{w}}}=\frac{{\int }_{V}{\boldsymbol{p}}\,{V}_{f1}dV}{{\int }_{V}{V}_{f1}dV}.$$

**Figure 4:**
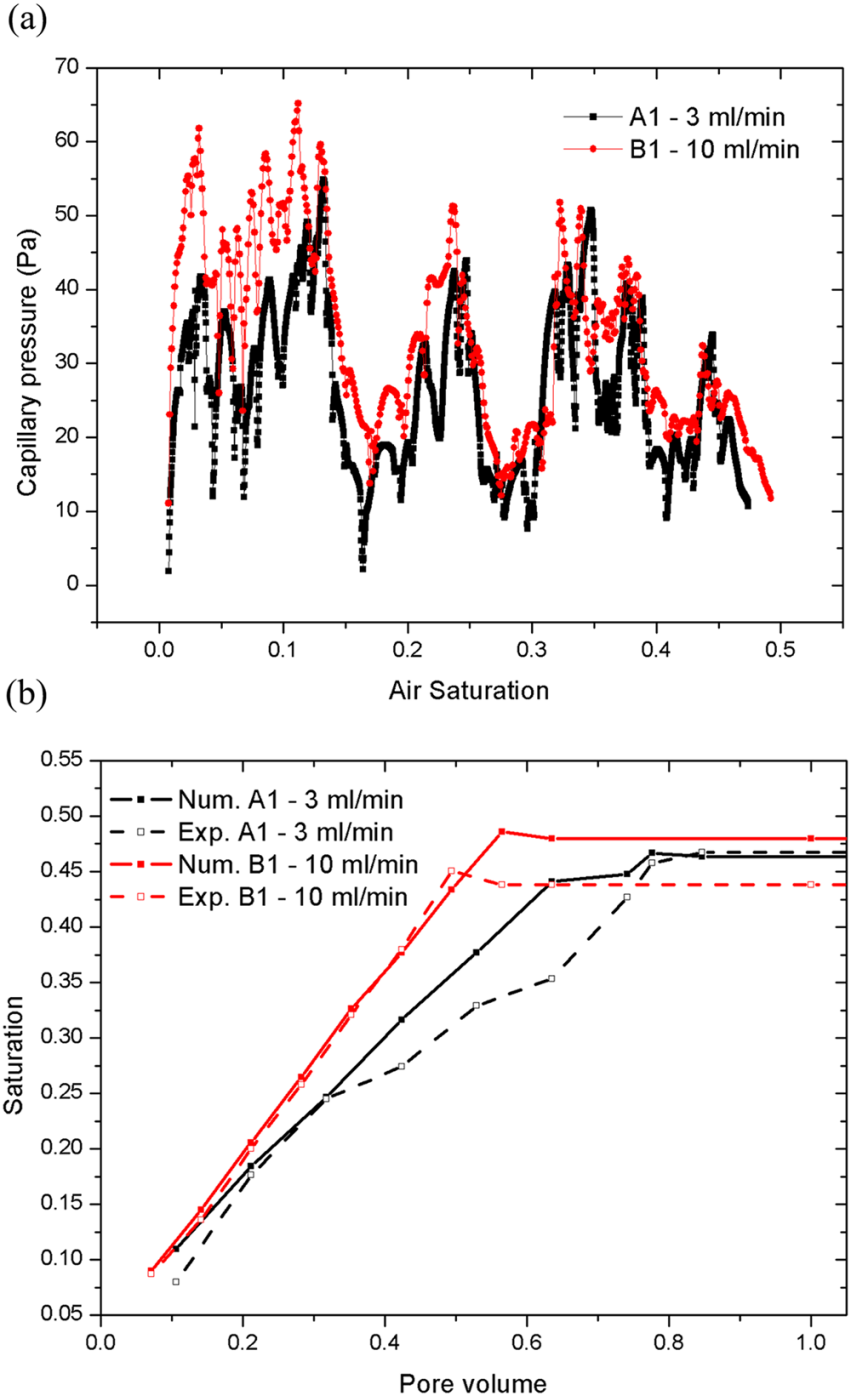
Effects of changing air saturation. (**a**) Macro-scale capillary pressure as a function of air saturation for different cases; and (**b**) comparison of the air saturation evolution from the tests and numerical simulations at *d*_*z*_ = 1 mm.

As shown by Fig. 4(b), the gas saturation increases as the injected gas volume increases during the initial drainage stage. In Fig. 4(a), the fluctuating section of capillary pressure curve represents the main drainage period, and the invading phase principally enters the porous model at all pressure ranges. Although the capillary pressure does not change much at this stage, the saturation of the invading phase increases rapidly. In the displacement process, the inlet pressure of invading phase air is kept constant by extracting gas into the porous section at a steady flow rate, and so the outlet pressure controls the entire displacement process. As the air enters, the surface deformation of water causes an air-water interface surface tension, creating a pressure jump at the interface which causes the water and air to move.

During displacement, the outflow rate of displaced phase water is stable. As the air continues to enter, when the pressure of invading phase air is larger than the valve pressure of all openings connected to the pores, the air passes immediately and moves into the next connected pores. As air continues to be extracted, water gradually flows out of the outlets until air reaches the stable saturation state and the capillary pressure decreases to almost zero. From Fig. 3, the numerical simulations of flow paths and two-phase air-water interface motion in the porous section accurately replicate the experimental observations. Quantitative comparisons of air saturation as a function of pore volume, are shown in Fig. 4(b) for various cases. It is shown that the air saturation increases with the injection volume of air. The overall velocity of fluid displacement in the simulations is slightly higher than that in the experiment, which may be because the impact of interface friction were not taken account in the simulation^6,47^. The numerical simulations and experimental observations also found that there exists an observable outlet effect wherein water reflows into some porous channels occupied by air after air reaches the outlets, which leads to the final saturation decrease of invading phase air. This can be seen in Fig. 4(b). where air saturation drops slightly after the breakthrough time and must been considered in the multi-phase flow in oil and gas mining engineering.

## Discussion

**Figure 5:**
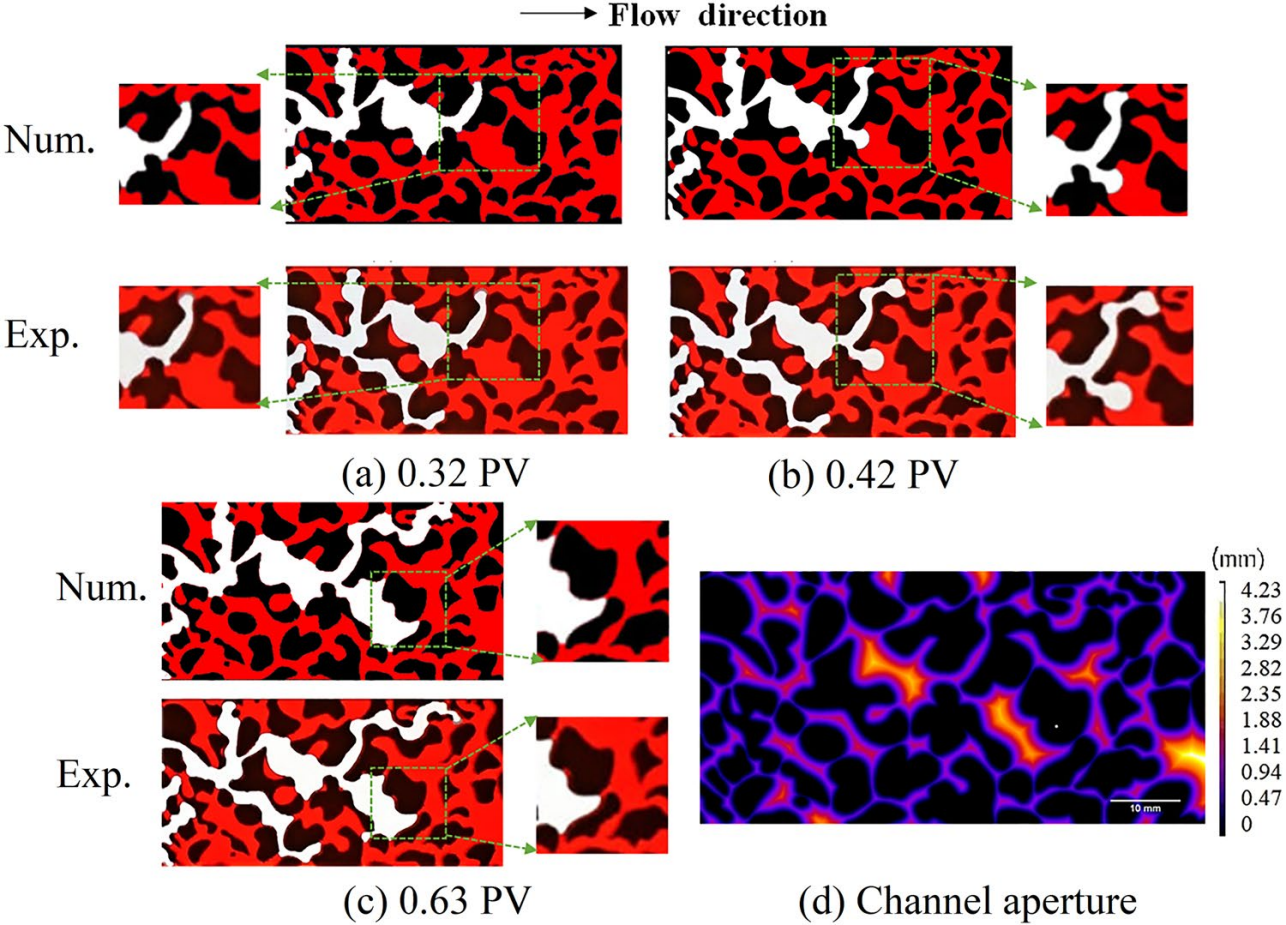
Experimental observations and numerical simulations. (**a**–**c**) Different stages of menisci propagation of selected sections along the time line for model A1. (**d**) Geometry of the porous model, with different colors (from purple to white) indicating the channel aperture. White areas represent the invading phase (air) and red areas represent the displaced phase (water). The solid phase is shown in black.

The experimental observations and numerical simulations show that the air first invades the wide branch channels, despite the existence of small pores downstream. Then, after a certain arrest time, the air invades the narrow channels. The meniscus evolution in the Y-shape pore junctions, shown in Fig. 5, shows that meniscus arrest exists during the displacement process in asymmetric pore junctions. This phenomenon was previously discussed in work by Sadjadi *et al*.^48^. For the wetting phase air displacing wetting phase water, the Laplace pressure in the narrow channel ***P***_***L***,***n***_ is larger than the Laplace pressure in the wide channel ***P***_***L***,***w***_. This is given by

3 $${{\boldsymbol{P}}}_{{\boldsymbol{L}},{\boldsymbol{n}}}=-\,2{\boldsymbol{\sigma }}\,\cos \,{\rm{\theta }}(\frac{1}{{{\rm{w}}}_{{\rm{n}}}}+\frac{1}{{\rm{\delta }}})\, > -\,2{\boldsymbol{\sigma }}\,\cos \,{\rm{\theta }}(\frac{1}{{{\rm{w}}}_{{\rm{w}}}}+\frac{1}{{\rm{\delta }}})={{\boldsymbol{P}}}_{{\boldsymbol{L}},{\boldsymbol{w}}},$$

where *w*_*n*_ and *w*_*w*_ are the width of narrow and wide channels, respectively. Therefore, during air-water displacement process in an asymmetric Y channel with the thinner width is filled first because it has the lower Laplace pressure. It was also found that the arrested air does not pass through some narrow channels until the breakthrough time because the pressure gradient is not high enough, as shown in Fig. 5(c).

**Figure 6:**
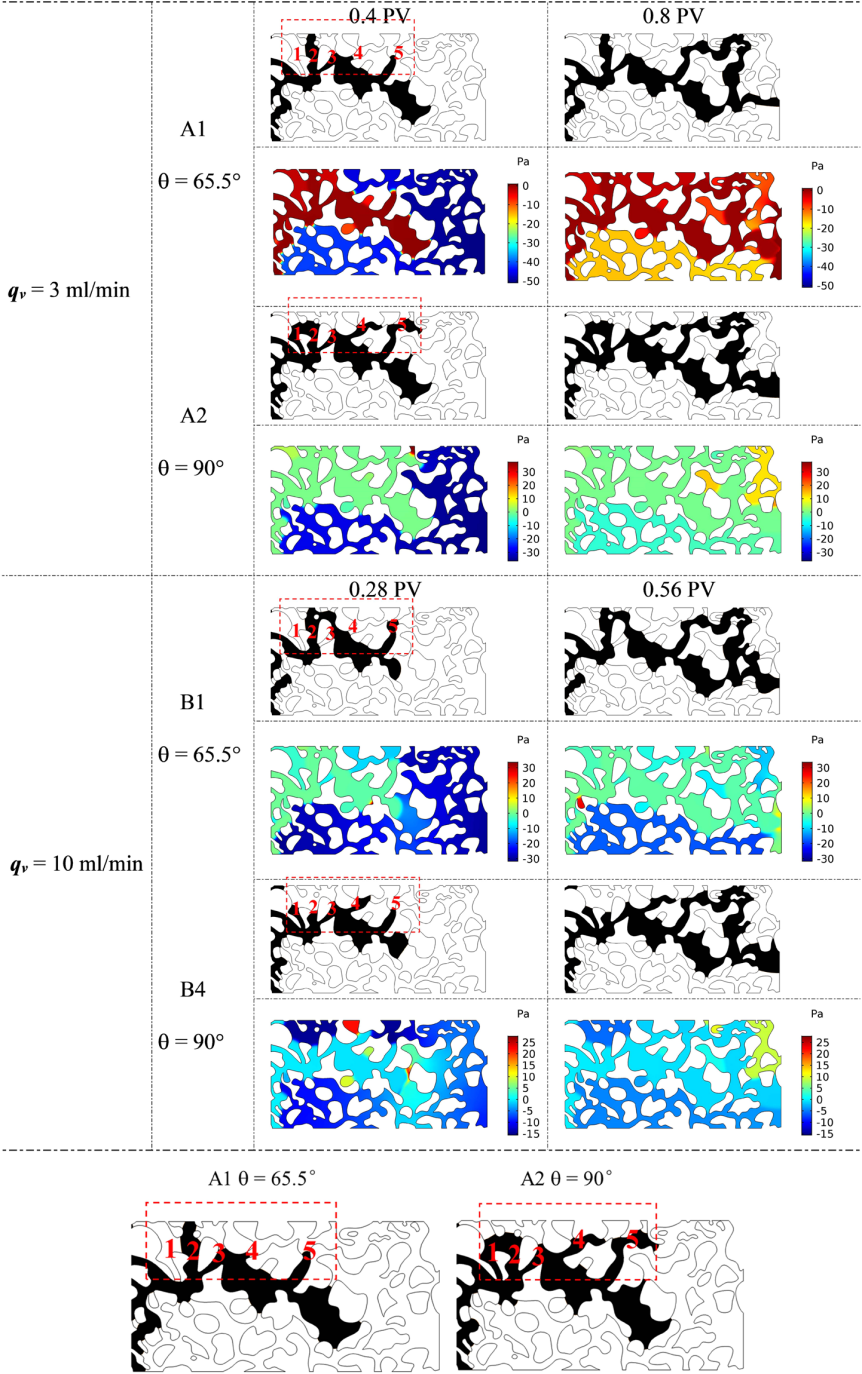
Comparison of the air-water two-phase flow paths and pressure distribution of A1, A2, B1, and B4 with different wetting conditions; the regions in red rectangles represent the path difference between two cases.

However, for θ = 90°, intermediate-wet media, cosθ = 0 and thus the Laplace pressure ***P***_***L***_ = 0, and the flow is determined by the displacing pressure. The air flow enters the branch channels simultaneously, as shown in Fig. 6. When θ = 65.5°, air first enter channel 2 with a larger pore, then flows into channel 3 with a smaller pore after some arrest time, and forms the eventual path in channel 3. While for θ = 90°, air breaks through more branch channels, and flow into 1, 2 and 3 simultaneously, but also form the eventual path in channel 3 due to the endpoint branches 1 and 2. The same path selection could be seen in the branch channels 4 and 5. The pressure distributions from these two cases have obvious differences, which is in accord with the flow path selection. In summary, during air-water displacement process, the migration path of two-phase interface is determined by displacing pressure and pore distribution in the branch channels.

**Figure 7:**
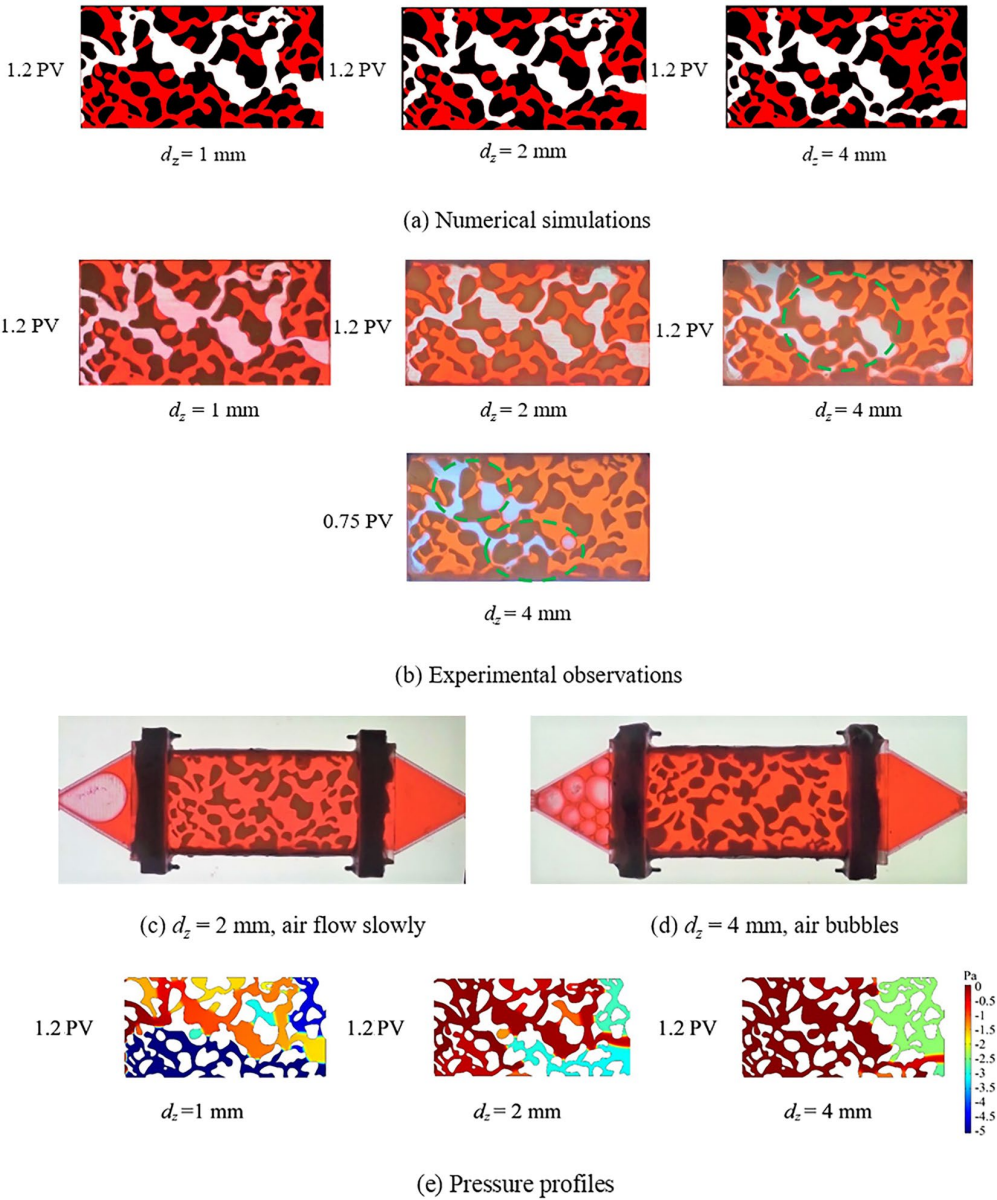
Comparison of two-phase distribution and pressure profiles from three different channel thickness at *q*_*v*_ = 10 ml/min: (**a**) Numerical simulation; (**b**) Experimental observation; (**c**) B2, *d*_*z*_ = 2 mm, air slowly flow into buffer zone in the experiments; (**d**) B3, *d*_*z*_ = 4 mm, air bubbles at the inlet in the experiment; (**e**) Pressure profile from the simulations. White color represents the invading phase (air), and red color represents the displaced phase (water). The solid phase is shown in black.

Figure 7 plots the comparisons of two-phase distribution and pressure profiles from three different channel thickness at ***q***_***v***_ = 10 ml/min as examples. From Fig. 7(a,b), it can be seen that the final phase distributions from three cases from numerical simulation are in accord with the experimental observations. The flow takes the second path, leading to a different fluid distribution with another two cases, when the channels thickness increase to 4 mm (approximately equal to the maximum size of pore throat 4.23 mm in this model). From the experimental phenomenon in Fig. 7(c,d), it was also found that when channel thickness is 4 mm, the air flows into buffer zone in the form of bubbles as the channel thickness rises, and air flow becomes discontinuous. This phenomenon may be because the channel thickness reaches to the diameter of air bubbles, about 2 mm^49^. However, such phenomenon could not be simulated in numerical works using the FEM phase method. That is, the phase field method with shallow channel approximate using the FEM in this study is no longer appropriate for simulating the two-phase fluid distribution in porous structures when the channel thickness approaches the diameter of air bubbles, although it could still track the flow paths reasonably.

For a representative element volume (REV), the flow channels can be regarded as rectangular channels in this study. The frictional resistance factor in flow channels can be calculated as^50^

4 $$f=\frac{\varDelta {\boldsymbol{p}}}{l}\frac{2{D}_{h}}{\rho {{\boldsymbol{u}}}^{2}},$$

where $\varDelta p$ refers to the pressure drop between inlets and outlets, Pa; *ρ* is the fluid density, kg/m^3^; ***u*** is the fluid velocity; *D*_*h*_ is the hydraulic diameter, and is expressed as, for rectangular channel,

5 $${D}_{h}=\frac{2{d}_{z}w}{{d}_{z}+w},$$

here, *d*_*z*_ is the channel thickness, mm; *w* is the channel width, mm. According to the Eqs (19) and (20), the frictional resistance factor *f* grows with increasing channel thickness *d*_*z*_. Therefore, the flow paths alter gradually, and the final saturation of invading air phase also decreases correspondingly. When the driving pressure is greater than the critical capillary pressure at the pore junctions, air will break through the pore throat. Once this occurs, the capillary pressure is released, and air enters the next pore channel. The capillary pressure grows along with air flow into the next pore junction. The capillary pressure fluctuates over time. The resistance to flow increases with the hydraulic diameter; therefore, air cannot break through some pore junctions (for example, ② and ③ in Supplementary Fig. S1). As a result, it breaks through some alternative flow paths with increasing channel thickness (see ① at *d*_*z*_ = 4 mm in Supplementary Fig. S1). In summary, the growth of resistance to flow is the main reason for the formation of secondary flow paths with increasing channel thickness in porous structures.

Our results indicate that the numerically simulated flow distributions are in agreement with the preferential flow paths from the experimental observations. The flow velocity and pressure profiles could quantitatively reflect the flow paths, and the streamlines are a good indicator for use in tracking preferential paths. Based on our experimental observations and numerical simulations, it can be concluded that air-water two-phase flow in porous structure has obvious preferential flow and fingering phenomenon. During air-water displacement process, the migration path of two-phase interface is determined by displacing pressure and pore distribution in the branch channels. For wet structures (θ < 90°), there exists the meniscus arrest during the displacement process in asymmetric pore junctions and narrow channels. Under a certain flow rate, the air invades first into the wide paths at Y-shape pore junctions, and after a certain arrest time also invades the narrow branch channels. For intermediate-wet structures (θ = 90°), the flow is driven by displacing pressure, and air flows into branch channels simultaneously and breaks through more branch channels. However, the air cannot pass through some tight channels because of weak pressure gradients. Such phenomenon aligns with the theoretical predictions. This study also found that there exists an obvious outlet effect wherein the wetting-phase water reflows into some porous channels occupied by invading phase air after air breaks through the outlets, which leads to the final saturation decrease of air and must been consider in the multi-phase flow in oil and gas mining engineering.

Overall, we found that thick channels can induce secondary air flow paths due to the increase in flow resistance, leading to a different fluid distribution compared with narrow channels. Therefore, channel thickness effects are not negligible in drainage displacement processes with air-water flow in porous structures. The secondary flow paths form as the channels thickness increase to almost the size of pore throat and the final air saturation reduces correspondingly. It was also found that air flows into porous structures in the form of bubbles as the channel thickness rising to approximate the size of air bubbles and air flow becomes discontinuous flow. However, such phenomenon could not be simulated in the numerical work using the FEM method, rendering the FEM technique inappropriate for modeling situations where the channel thickness approaches the size of air bubbles.

A reasonable agreement is shown between the experimental data and numerical simulations, which allows confirming the validity of model implemented in COMSOL for the simulation of flow patterns and paths tracking of two-phase fluid flow in the porous media. It was proved that the phase field model is able to handle the interface dynamics of air-water two-phase flow with larger density and viscosity ratios, and could satisfactorily predict the preferential paths in porous media. This study may provide the reference for studies on multi-phase flow and preferential paths in heterogeneous porous structures.

## Methodology and Models

### Experimental observations during the drainage displacement process in porous structures

In order to explore the fundamental process underlying flow paths and to visualize two-phase fluid motions in porous structures, we used a model to examine the effect that pore structure characteristics have on the air-alternation-water process. In this study, the porous structures we used is derived from a series of CT micrographs of a Berea sandstone based on the original work of Sirivithayapakorn *et al*.^16^ and Auset *et al*.^17^ (see Supplementary Fig. S2). This pore structure is very typical and has been used in published experimental^17^ and numerical work^6^. It has several main flow pathways and adequate pore connectivity. Moreover, this pore structure has the heterogeneity of real pore structures, and simplifies the complexity of pore structures at sub-pore scales. These pore characteristic makes it easy to test the numerical models, or explore the multiphase flow mechanism in porous media. This study is mainly concerned with the influence of pore morphology on flow, regardless of the matrix properties, etc. The minimum channel aperture in the model is about 0.16 mm and thus the experimental model was produced using 3D printing technology that allows creating transparent patterns that have dimensions in the range of those existing at this scale. The model has a porous body of *d*_*z*_ thick in the middle and splints of 2 mm thick on both sides. The completely transparent photosensitive organic polymer resin was used for the pore model. Due to the transparency of this material, the flow process in the pore body can be visually observed. In the micromodel, we enlarged the pore size to millimeter scale to make it easier to observe the flow process. The void space was filled with water, and water was mixed with red ink to track the flow (see water-saturated model in Supplementary Fig. S2). As in some of our previous studies^32^, an experimental setup with an imaging system was used for each drainage displacement experiment (see Supplementary Fig. S2). The micromodel was placed horizontally on a white-light-emitting board to enhance the imaging contrast. The model inlet was connected to a well-controlled syringe pump for water injection and withdrawal. The outlet was in contact with the atmosphere under room conditions. A high-speed camera was assembled above the micromodel against a scope stage adjustable in three directions. During the experiment, the camera height was fixed with the stage, while the horizontal position was adjusted for optimal observation of the mobile air-water interface. The dimensions of each part of micromodel can be seen in Supplementary Fig. S2.

Before the drainage experiment, distilled water was dyed with red ink for superior phase discernment, which means that the dyed water was used as a tracer to distinguish between the two-phase fluids (air and water) distribution for experimental observations in the displacement process. This was then injected into the porous model at a low rate to establish a fully water-saturated condition with no trapped air bubbles (see Supplementary Fig. S2)^32^. The drainage experiments were carried out at room temperature. After the above preparatory steps were completed, the drainage experiments were conducted by withdrawing the syringe pump at a constant flow rate ***q***_***v***_. In this study, two flow rates ***q***_***v***_ (=3 ml/min and 10 ml/min) and three channel thickness *d*_z_ (=1 mm, 2 mm and 4 mm) were designed to investigate the fluid flow in these porous structures. The capillary number and mobility ratio were calculated using the equations^6^

6 $${\rm{Ca}}=({\mu }_{air}\times {\boldsymbol{v}})/({\boldsymbol{\sigma }}\,\times \,\cos \,{\rm{\theta }})$$

and

7 $${\rm{M}}={\mu }_{air}/{\mu }_{w}.$$

Here, ***v*** is the Darcy velocity; *μ*_*air*_ and *μ*_*w*_ are the viscosity of air and water, respectively; and **σ** (=0.072 N/m) and θ are the interfacial tension and air-water contact angle, respectively. Lenormand *et al*.^6,51^ discussed how the drainage displacement processes can be characterized by means of the capillary number. Referring to the previous research^7,52^, for low capillary numbers (commonly assumed to be less than 10^−5^), flow in porous media is dominated by capillary forces whereas for high capillary numbers the capillary forces are negligible compared to the viscous forces. Here, the air–water displacement, with a minimum capillary number of log Ca = −4.89 and a maximum of log Ca = −4.38, and a mobility ratio of log M = −1.74, fall within the crossover from capillary to viscous fingering and the viscous fingering regimes, respectively. Tests were repeated two times having identical flow rate under the same testing condition to obtain stable results and reduce data discreteness. The sketch of fluid flow regimes observed in the micromodel are shown in the results sections. This observation exhibits the entire drainage displacement process and its characteristics in porous model.

### Model for drainage displacement in water saturated porous media

A numerical model was built in COMSOL’s built-in application modes to predict and analyze the preferential paths of drainage experiments described in the previous section. We simulate the displacement of water by air in a two-dimensional pore geometry using the phase field method for capturing the air-water interface when solving the Navier–Stokes equations. The laminar two-phase flow, phase field interface is used to model two fluids separated by a fluid interface and where the moving interface is tracked in detail, taking into account surface tension forces and wetting effects. The interface position is tracked by solving two additional transport equations, one for the phase field variable and one for the mixing energy density. The interface movement is determined by the minimization of free energy. The fluids are considered to be compressible and Newtonian fluids, and the system is assumed to be isothermal. The properties of air and water are assumed to be constant, and the effect of gravity on flow and transport is considered. One of the strengths of this model is has proven accurate and has the ability to handle the interface dynamics of two-phase flow with larger density and viscosity ratios using the Cahn-Hilliard equation.

### Phase field method

The phase field method, using the Cahn-Hilliard equation, was employed to track the diffuse interface separating the immiscible phases, water and air. This is expressed by^53,54^

8 $$\frac{\partial \varnothing }{\partial t}+{\boldsymbol{u}}\cdot {\nabla }\varnothing ={\nabla }\cdot \frac{\gamma \lambda }{{\varepsilon }^{2}}{\nabla }\varPsi $$

and

9 $$\varPsi =-\,{\nabla }\cdot {\varepsilon }^{2}{\nabla }\varnothing +({\varnothing }^{2}-1)\varnothing .$$

Where the diffuse interface is the region where the dimensionless phase field variable ∅ goes from −1 to 1. Furthermore, ∅ is the phase field parameter, ***u*** is the fluid velocity (m/s), γ refers to the mobility (m^3^·s/kg), λ is the mixing energy density (N), and ε (m) is the interface thickness parameter. The *Ψ* variable is the phase field help variable. The surface tension **σ** is calculated by^6^:

10 $${\boldsymbol{\sigma }}=\frac{2\sqrt{2}}{3}\cdot \frac{\lambda }{\varepsilon },$$

11 $$\varepsilon =\frac{{h}_{c}}{2},\,{\rm{and}}$$

12 $$\gamma ={\varepsilon }^{2},$$

where *h*_*c*_ is the characteristic mesh size in the region passed by the interface. The volume fractions of the two-phase fluids are^6^

13 $${V}_{f1}=\frac{1-\varnothing }{2}\,\,{\rm{and}}\,{{\rm{V}}}_{f2}=\frac{1+\varnothing }{2}.$$

In this model, water is fluid 1 and air is fluid 2. The density *ρ* (kg/m^3^) and viscosity *μ* (Pa·s) of the mixture are assumed to vary smoothly over the interface by letting^6^

14 $${\rm{\rho }}={{\rm{\rho }}}_{w}+({\rho }_{air}-{\rho }_{w}){{\rm{V}}}_{{\rm{f}}2}$$

and

15 $$\mu ={{\rm{\mu }}}_{w}+({\mu }_{air}-{\mu }_{w}){{\rm{V}}}_{{\rm{f}}2},$$

where *ρ*_*air*_ and *ρ*_*w*_ denote the density of water and air, respectively.

### Mass and momentum transport

The compressive Navier-Stokes equation with surface tension and gravity was used to describe the transport of mass and momentum for fluids. This is expressed as^55^

16 $$\rho \frac{\partial {\boldsymbol{u}}}{\partial t}+\rho ({\boldsymbol{u}}\cdot \nabla ){\boldsymbol{u}}=-\,\nabla {\boldsymbol{p}}+\nabla \cdot ({\rm{\mu }}(\nabla {\boldsymbol{u}}+{(\nabla {\boldsymbol{u}})}^{{\boldsymbol{T}}})-\frac{2}{3}\mu (\nabla \cdot {\boldsymbol{u}}){\bf{I}})+{{\boldsymbol{F}}}_{st}+{{\boldsymbol{F}}}_{\mu }+\rho {\boldsymbol{g}}$$

and

17 $$\nabla \cdot {\boldsymbol{u}}={\bf{0}},$$

where, *ρ* denotes the density (kg/m^3^), *μ* equals the dynamic viscosity (Pa·s), ***u*** represents the velocity (m/s), ***p*** denotes the pressure (Pa), and ***g*** is the gravity vector (m/s^2^). In addition, ***F***_***s****t*_ is the surface tension force acting at the air-water interface. This is computed as a distributed force over the interface,

18 $${{\boldsymbol{F}}}_{st}=G\nabla \varnothing ,$$

where *G* is the chemical potential (J/m^3^) given by^53^

19 $$G=\lambda [\,-\,{{\rm{\nabla }}}^{2}\varnothing +\frac{\varnothing ({\varnothing }^{2}-1)}{{\varepsilon }^{2}}]=\frac{\lambda }{{\varepsilon }^{2}}\varPsi .$$

Simple 2D models may produce inaccurate results for such flow problems because they often exclude the effect of channel thickness on the flow and assume that the channel thickness is much smaller than the channel aperture^42,56^. Here, the used model takes such effects into account by adding a drag term as a volume force to the momentum equation,

20 $${{\boldsymbol{F}}}_{\mu }=\,-12\frac{\mu {\boldsymbol{u}}}{{{d}_{z}}^{2}},$$

where *d*_*z*_ is the channel thickness, and is set as three levels to consider the channel thickness effect on flow. This term represents the resistance that parallel boundaries impose on the flow; however, it does not account for any changes in velocity due to variations in the cross-sectional area of the channels.

### Boundary conditions

The model is initially filled with water, so the initial volume fraction of water is 1 here. Air flows from inlet to outlet across the geometry. Due to that water is pumped out of the outlets at a constant flow rate. The flow rate is set as a constant at the outlet and the volume fraction of air is 1 at the inlets in the simulation. The wetted wall boundary condition is used for solid walls in contact with fluid interfaces. Along a wetted wall the contact angle for the fluid, θ is specified and across it, the mass flow is zero. This is prescribed by^53^

21 $${\bf{n}}\cdot {\varepsilon }^{2}\nabla \varnothing ={\varepsilon }^{2}\,\cos \,{\rm{\theta }}|\nabla \varnothing |,$$

22 $${\bf{n}}\cdot \frac{\gamma \lambda }{{\varepsilon }^{2}}{\rm{\nabla }}\varPsi =0,$$

where the contact angle θ, that is, the angle between the wall and the fluid interface (see Supplementary Fig. S2) is set as 65.5° measured from tests.

### Determination of material parameters

The domain of interest covers 92 mm by 40 mm and the thickness is approximately 1/2/4 mm. The fluids in pores do not penetrate into the solid part (black area in Supplementary Fig. S2). Six cases (A1-A2, B1-B4) with the same conditions as the experimental tests are used to simulate the drainage experiments described in the previous section. Cases A2 and B4 were carried out to study the influence of wetting conditions (wetting angle) on two-phase flow. A time-dependent solver is used in the simulation since the position of an interface is almost always dependent on its history. Table 1 summarizes the basic data and fluid properties used in the simulation.

**Table 1:** Parameters for case studies.

No.	*q*_*v *_(ml/min)	*d*_*z*_ (mm)	*μ*_*w*_ (×10^−3^ Pa·s)	*μ*_*air*_ (×10^−5^ Pa·s)	*ρ*_*w*_ (kg/m^3^)	*ρ*_*a*_ (kg/m^3^)	log Ca	log M	θ (^ο^)	σ (×10^−2^ N/m)
A1	3	1	1	1.81	1000	1.28	−4.89	−1.74	65.5	7.21
A2		1							90	
B1	10	1	1	1.81	1000	1.28	−4.38	−1.74	65.5	7.21
B2		2								
B3		4								
B4		1							90	

## Supplementary information

Supplementary Information
