# Supplementary material for: Preferential Paths of Air-water Two-phase Flow in Porous Structures with Special Consideration of Channel Thickness Effects
Source: Sci Rep. 2019 Nov 7;9:16204. doi: 10.1038/s41598-019-52569-9 (PMC6838087; doi:10.1038/s41598-019-52569-9)
Supplement: Supplementary file 1 — Supplementary Information [file 41598_2019_52569_MOESM1_ESM.docx]

**Supplementary Information**

# **Preferential Paths of Air-water Two-phase Flow** **in Porous Structures with Special Consideration of Channel Thickness Effects**

Jinhui Liu ^a^, Yang Ju ^b, c *^, Yingqi Zhang ^d^, Wenbo Gong ^a^

*^a^* *School of Mechanics and Civil Engineering,* *China University of Mining and Technology, Beijing, 100083, China*

*^b^ State Key Laboratory of Coal Resources and Safe Mining, China University of Mining and Technology, Beijing, 100083, China*

*^c^ Frontier Scientific Research Centre for Fluidized Mining of Deep Underground Resources, China University of Mining & Technology, 1 University Ave, Xuzhou 221006, China*

*^d^ Energy Geosciences Division, Lawrence Berkeley National Laboratory, Berkeley, California, CA**94720, USA*

*** Corresponding author:** Yang Ju, PhD.,

China University of Mining & Technology at Beijing

D11 Xueyuan RD 100083, Beijing, P. R. China

Tel: +86 10 62331490; Fax: +86 10 62331253

Email: [juy@cumtb.edu.cn](mailto:juy@cumtb.edu.cn)

Supplementary figures


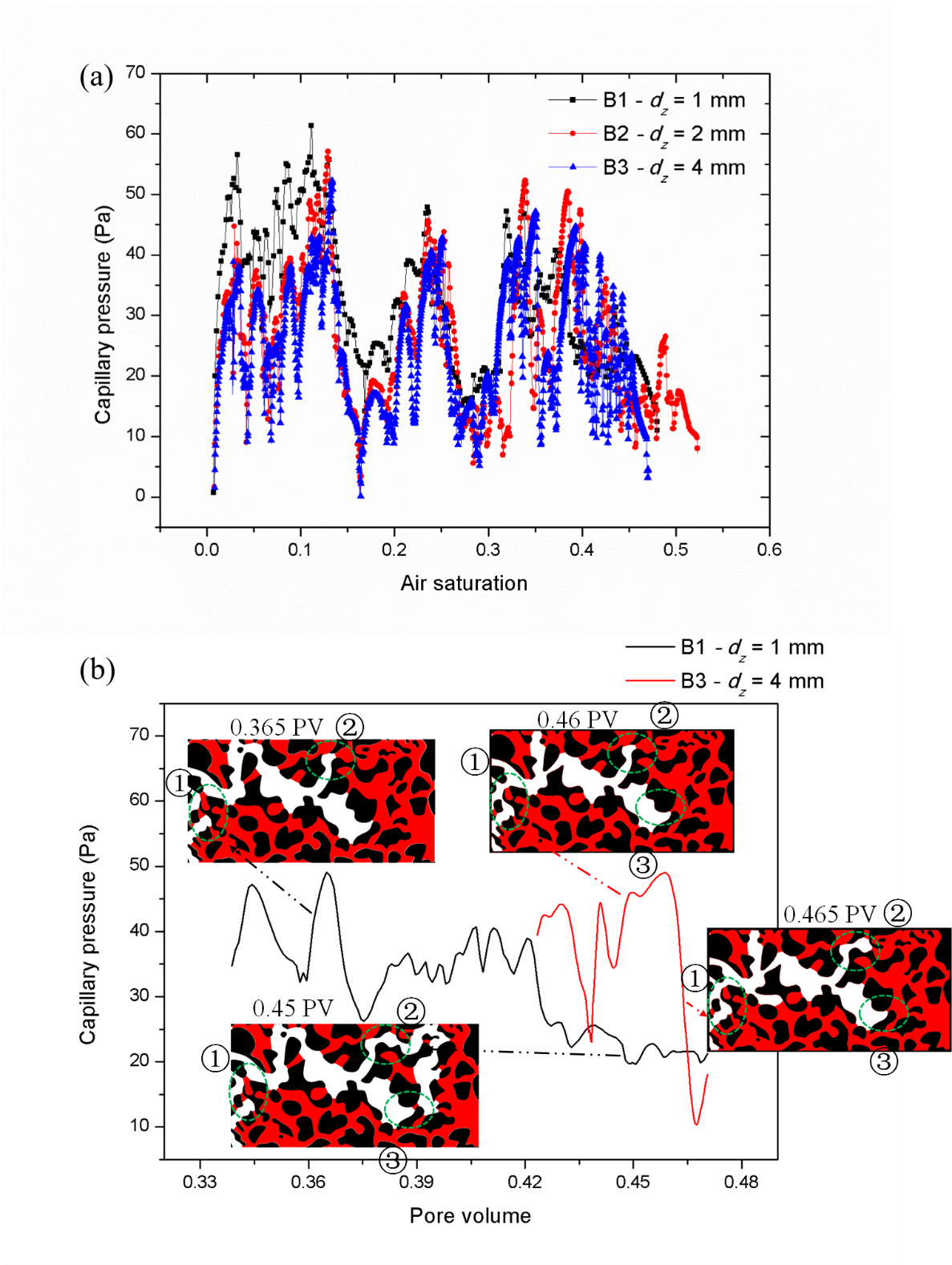


Figure S1 (a) macro-scale capillary pressure as a function of air saturation for different cases; (b) capillary pressure variation with the breakup through pore throats


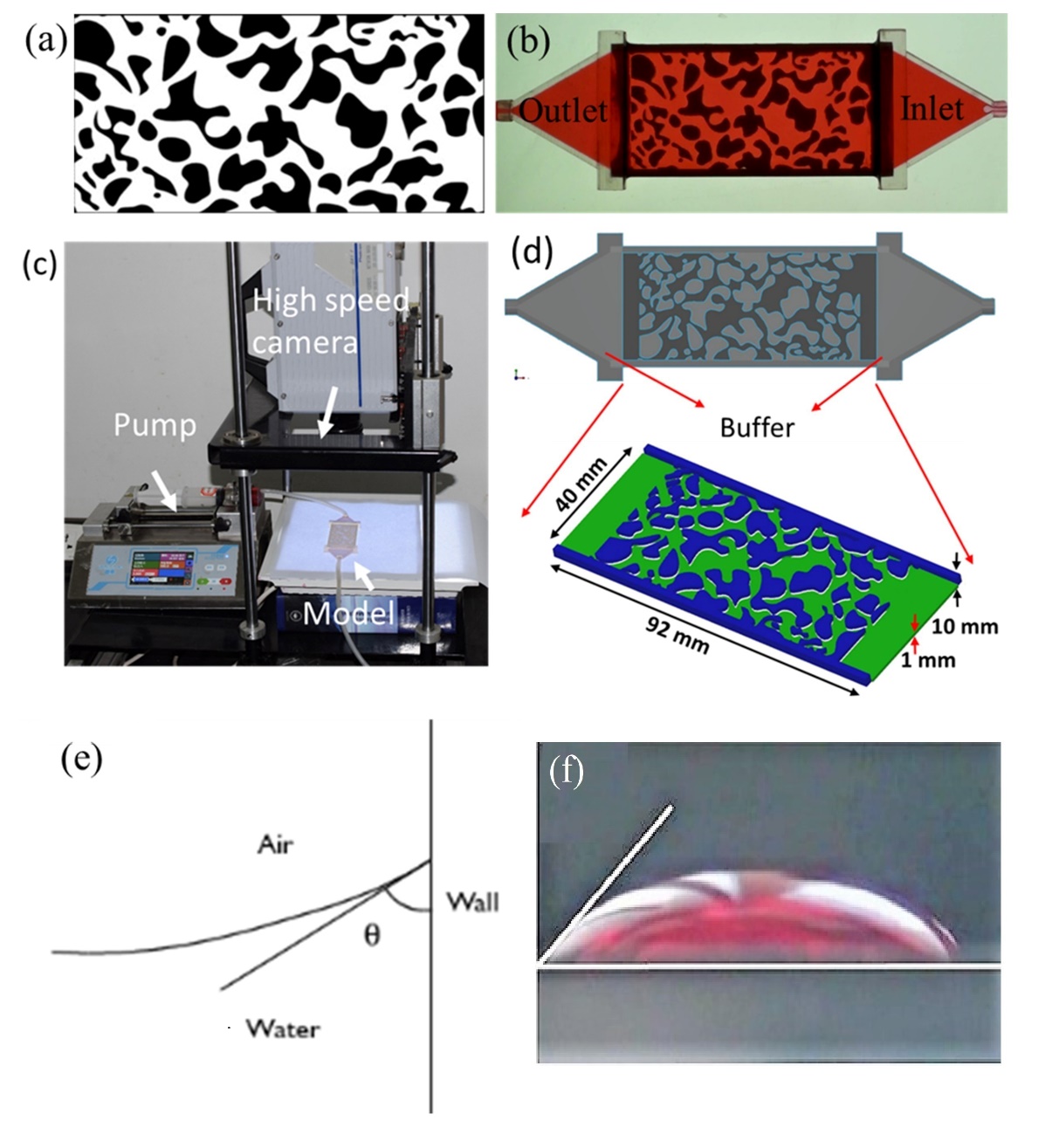


Figure S2 (a) original image of porous structures (the white areas are continuous pores and throats, whereas the black areas are the matrix); (b) saturated water model; (c) experimental setup; (d) dimensions of printing model; (e) contact angle definition; (f) contact angle measurements and computation of dyed water on printing plate.
